# Gelatin-Based Microspheres of Ciprofloxacin for Enhanced Lung Delivery and Biofilm Eradication in *Pseudomonas aeruginosa* Pulmonary Infections

**DOI:** 10.3390/gels11080567

**Published:** 2025-07-23

**Authors:** Luis Monrreal-Ortega, Rocío Iturriaga-Gallardo, Andrea Vilicic-Rubio, Pedro Torres, Patricio Leyton, Javier O. Morales, Tania F. Bahamondez-Canas, Daniel Moraga-Espinoza

**Affiliations:** 1 Escuela de Química y Farmacia, Facultad de Farmacia, Universidad de Valparaíso, Valparaíso 2340000, Chile; luis.monrreal@postgrado.uv.cl (L.M.-O.); rocio.iturriaga@alumnos.uv.cl (R.I.-G.); andrea.vilicic@postgrado.uv.cl (A.V.-R.); 2 Laboratory of Drug Delivery, Departamento de Ciencias y Tecnología Farmacéuticas, Facultad de Ciencias Químicas y Farmacéuticas, Universidad de Chile, Independencia 8380494, Chile; pedro.torres@ug.uchile.cl (P.T.); jomorales@ciq.uchile.cl (J.O.M.); 3 Advanced Center for Chronic Diseases, Facultad de Ciencias Químicas y Farmacéuticas, Universidad de Chile, Independencia 8380494, Chile; 4 Center of New Drug for Hypertension and Heart Failure (CENDHY), Facultad de Ciencias Químicas y Farmacéuticas, Universidad de Chile, Independencia 8380494, Chile; 5 Instituto de Química, Pontificia Universidad Católica de Valparaíso, Valparaíso 2340025, Chile; patricio.leyton@pucv.cl; 6 Centro de Investigación, Desarrollo e Innovación en Productos Bioactivos (CInBIO), Universidad de Valparaíso, Valparaíso 2340000, Chile

**Keywords:** gelatin, microspheres, dry powder inhalers, biofilms, pulmonary delivery, chronic lung infections

## Abstract

Chronic lung infection is the main predictor of morbidity and mortality in cystic fibrosis (CF), and current pharmacological alternatives are ineffective against *Pseudomonas aeruginosa* infections. We developed ciprofloxacin (CIP) for inhalation, aiming at improving its solubility through the formation of an amorphous solid dispersion (ASD) using gelatin (GA). CIP and GA were dissolved in varying ratios and then spray-dried, obtaining CIP-GA microspheres in a single step. The dissolution rate, size distribution, morphology, and aerodynamic properties of CIP-GA microspheres were studied, as well as their antimicrobial activity on *P. aeruginosa* biofilms. Microspheres formulated with a higher GA ratio increased the dissolution of CIP ten-fold at 6 h compared to gelatin-free CIP. Formulations with 75% GA or more could form ASDs and improve CIP’s dissolution rate. CIP-GA microspheres outperformed CIP in eradicating *P. aeruginosa* biofilm at 24 h. The spray-drying process produced CIP-GA microspheres with good aerodynamic properties, as indicated by a fine particle fraction (FPF) of 67%, a D_50_ of 3.52 μm, and encapsulation efficiencies above 70%. Overall, this study demonstrates the potential of gelatin to enhance the solubility of poorly soluble drugs by forming ASDs. As an FDA-approved excipient for lung delivery, these findings are valuable for particle engineering and facilitating the rapid translation of technologies to the market.

## 1. Introduction

Cystic fibrosis (CF) is an autosomal recessive disease that affects 85,000–100,000 people worldwide and leads to death in 90% of patients [1,2]. The incidence appears to be decreasing and is currently estimated to be between 1/3000 and 1/6000, with patients reaching a survival of nearly 50 years [3]. The characteristic thick and sticky mucus of CF patients plays a big role in this outcome, as it becomes an ideal environment for opportunistic *Pseudomonas aeruginosa* infections. These infections have been recognized as one of the main predictors of morbidity and mortality in CF [4,5,6]. Therefore, this study aimed to develop a gelatin-based delivery platform to enhance the dissolution rate of a pH-dependent antibiotic by formulating it as a dry powder for inhalation. This approach leverages gelatin type A (GA)—an FDA-approved excipient already used in high doses (up to 100 mg) in inhalable capsule formulations—as both a stabilizing polymer and a matrix former for amorphous solid dispersions (ASDs). By integrating gelatin into the formulation, the goal is to improve drug solubility and stability, thereby enabling fast-tracked development of inhaled therapies for chronic lung infections [7,8,9]. The increased dissolution rate aims to make the antibiotic readily available for treating *P. aeruginosa*-associated infections in CF patients.

Current treatment options are mainly ineffective against *P. aeruginosa* infection due to its remarkable resistance mechanisms [6,10]. The most challenging mechanism to overcome is the deployment of a polypeptide, mucus-like barrier that protects the bacterial community. This aggregated life form is commonly known as a biofilm [10,11]. Oral and parenteral routes are inefficient for delivering therapeutic concentrations to the lungs, as the drug is distributed throughout a large volume before reaching the site of infection. Consequently, higher doses are required, exacerbating systemic side effects [12]. In contrast, while inhaled antibiotics provide more effective drug delivery to the lungs, currently available options on the market are nebulized therapies (Cayston^®^, aztreonam 75 mg per vial (Gilead Sciences Inc., Foster City, CA, USA); Quinsair^®^, levofloxacin 240 mg per ampoule (Chiesi Farmaceutici SpA., Palermo, Italy), for which patients have poor compliance, and the other half of the arsenal are dry powder inhalers (DPIs) (Tobi^®^, Podhaler 28mg per capsule, recommended dose 112 mg (Viatris Specialty LLC., Canonsburg, PA, USA) and Colobreathe^®^, colistimethate sodium 125 mg per capsule (Essential Pharma Ltd., Egham, Surrey, UK), which have shown inefficacy past the moderate stage of the disease [6,13]. Ciprofloxacin (CIP) is a fluoroquinolone characterized by its high potency and broad-spectrum activity. CIP inhibits the activity of subunit A of DNA gyrase enzymes (topoisomerase II and topoisomerase IV) [14]. It has garnered significant interest from researchers and pharmaceutical companies due to its efficacy against Gram-negative bacteria [15,16]. Since its appearance, CIP has had a storied background of efficacy against *Pseudomonas* spp.-related infections. For years, the high potency of this drug has been leveraged by administering high doses to compensate for its poor bioavailability and limited distribution to the lungs following oral administration. Therefore, the previous approach is restricted because, at these doses, dangerous side effects, such as tendonitis, blurred vision, and tinnitus, become prevalent [17].

Inhalable dry powder formulations of CIP have been investigated to deliver a more potent antibiotic through inhalation, aiming to enhance patient compliance in chronic lung infection treatment. However, progress in this area has been hindered by the drug’s inherent physicochemical limitations [6]. Though the formation of a hydrochloride form improves its aqueous solubility (from 0.0015 to 0.067 mg/mL at 20 °C), CIP still exhibits minimal aqueous solubility [18,19] at the lung’s pH of 7.4 due to its zwitterionic nature at this physiological pH [20]. At this point, the molecule contains two ionizable functional groups (a carboxylic acid and an amine) bearing opposite charges, leading to a net-zero charge and significantly reduced solubility [21].

Spray drying has been extensively investigated as a one-step technique for particle size reduction and the amorphization of dry powders in pharmaceutical applications. The amorphization of carrier-free CIP has shown promise in enhancing solubility. However, the resulting dry powders exhibit limited physical stability, as the amorphous CIP molecules rapidly reorganize into different polymorphic forms [22,23,24]. These polymorphs alter the particles’ aerodynamic properties and lead to inconsistent aerosol deposition profiles, undermining the reliability and efficacy of the formulation for inhalation therapies. This behavior occurs because the dry powder has high free energy due to the energy introduced during the size reduction process and the interactions between the oppositely charged groups of CIP molecules. Co-spray-drying CIP with excipients in solution has been utilized to address this issue. The presence of polymeric excipients enables interactions with CIP molecules, such as charge interactions or hydrogen bonding, which inhibit the drug’s tendency to self-interact and reorganize into crystalline states. By stabilizing CIP in its amorphous form, these excipients improve the physical stability of the dry powder formulation and enhance its suitability for consistent aerosolization and pulmonary delivery [7,24,25]. Additionally, depending on the specific polymer used, this approach can delay the release of CIP, mitigating rapid systemic absorption from the lungs. While advancements in this strategy have resulted in dry powders with excellent aerosol performance and physical stability, many of the excipients employed lack well-established safety profiles for lung delivery. This study addresses these limitations by formulating inhalable CIP microspheres using gelatin as a matrix-forming excipient. Unlike many traditional carrier polymers, gelatin is FDA-approved for high-dose inhalation, making it an excellent candidate for such applications [26]. Its capacity to be incorporated at high percentages in formulations enables the formation of ASD with CIP, significantly enhancing the drug’s dissolution rate [25].

Therefore, this study aimed to develop a spray-dried powder formulation of amorphized CIP using gelatin as a stabilizing carrier, with the goal of enhancing CIP solubility in the lung environment and enabling its potential use as an alternative orally inhaled antimicrobial therapy for chronic lung infections.

## 2. Results and Discussion

The formulations developed in this study aimed to address challenges such as the limited dissolution of CIP at lung pH levels. Additionally, it focused on reformulating CIP, which is traditionally administered as an oral tablet, into a form suitable for direct respiratory administration.

### 2.1. Encapsulation Efficiency (EE) of Spray-Dried Microspheres

**Figure 1:**
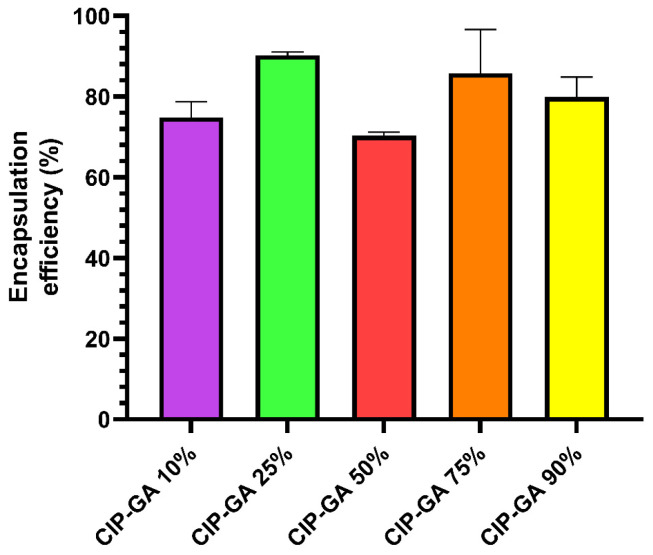
Encapsulation efficiency of CIP-GA microsphere formulations. Ciprofloxacin (CIP)-loaded gelatin A (GA) microspheres were prepared by spray drying, and the encapsulation efficiency was determined based on the theoretical CIP content, quantified by UV spectroscopy. Each formulation is named based on its CIP content (*n* = 3; mean ± S.D.).

CIP microspheres were obtained by spray-drying solutions of CIP and GA at different ratios. All inhalable dry powder formulations showed EE above 70%, with the CIP-GA 25% formulation reaching up to 90% (Figure 1). These values were used to calculate the actual dose delivered during the dissolution test and the antimicrobial activity.

### 2.2. Gelatin Encapsulation Increases CIP Dissolution

**Figure 2:**
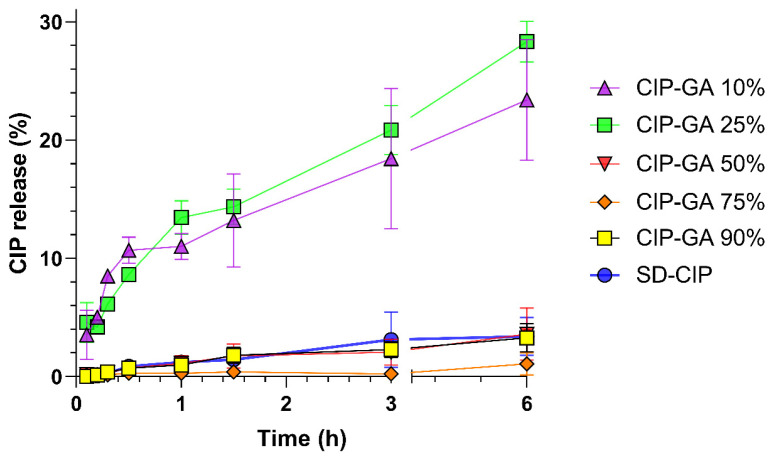
Dissolution profile of CIP-GA microspheres at varying CIP percentages. CIP release from CIP-GA microspheres compared to CIP dissolved from the GA-free spray-dried CIP (SD-CIP) using a Franz cell dissolution test at pH 7.4 (*n* = 4; mean ± S.D.).

CIP exhibited a higher dissolution rate when formulated as microspheres compared to spray-dried CIP (SD-CIP) (Figure 2). CIP-GA 25% (green squares) demonstrated the highest dissolution rate, releasing 14% and 29% of CIP at 1 and 6 h, respectively. This was closely followed by CIP-GA 10% (purple triangles), which released 11% and 23% at 1 and 6 h, respectively. The remaining formulations showed poor dissolution profiles, similar to that of SD-CIP (blue circles), with only 3% dissolved at 6 h, while the CIP-GA 75% formulation (orange diamonds) exhibited a significantly reduced dissolution, with just 1% dissolved at 6 h. 

The zwitterionic molecular structure of CIP makes it difficult to dissolve in certain body fluids, being soluble only at pH levels below 5 and above 10 [27]. Thus, the efficiency of CIP can be hampered if the environment in which it is administered is unsuitable for its dissolution. The current use of CIP as an antimicrobial relies on its rapid dissolution and absorption in an acidic environment when taken orally, even for treating lung infections. Therefore, enabling inhaled administration would be highly beneficial, especially since pulmonary infections are common in chronic diseases like CF. However, this goal is difficult to achieve due to CIP’s pH-dependent solubility.

The mucus in the lungs creates a slightly acidic to neutral environment (pH 6.8–7.4), significantly affecting the dissolution rate of CIP, even when it is amorphized through spray drying [28,29]. The results from the Franz cell study performed at pH 7.4 indicated a notable increase in the dissolution rate of CIP when using gelatin for microsphere formation by spray drying. 

Additionally, under our experimental conditions (acetate buffer at pH 4.5), both CIP and gelatin are expected to carry net-positive charges, minimizing the likelihood of ionic interactions prior to spray drying. This is consistent with the findings by Silva et al. (2018), who used distilled water (pH ~5.5–6.0) to dissolve CIP and gelatin, and reported no evidence of chemical interaction based on FTIR analysis [30]. Since our formulation pH is even lower, this electrostatic repulsion is likely more pronounced. Therefore, the enhanced dissolution observed in CIP-GA microspheres can be attributed to post-drying amorphization and matrix dispersion rather than pre-spray-drying drug–polymer interactions. These conclusions also align with our previous experience using gelatin for inhalable microparticle systems, where ionic interactions were observed with anionic cromolyn sodium and cationic ipratropium bromide in aqueous solution before spray drying due to their opposing charges [25]. In contrast, CIP and gelatin, both positively charged at pH 4.5, are unlikely to engage in such interactions. However, the long-term physical stability of the amorphous CIP form, particularly in the lead CIP-GA 25% formulation, remains to be confirmed and should be rigorously assessed before in vivo studies.

**Figure 3:**
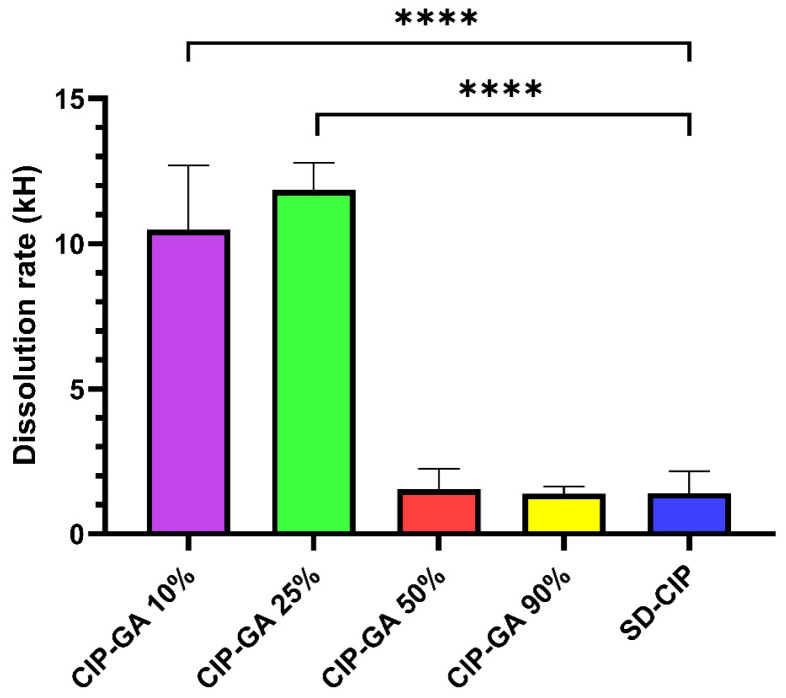
Dissolution rate (kH) of CIP-GA microsphere formulations in pH 7.4 buffer. The dissolution rate was calculated for matrix systems using the Higuchi model (*n* = 4; mean ± S.D.). Asterisks in brackets indicate a statistically significant difference (**** *p* < 0.0001) compared to the spray-dried pure drug.

The Higuchi dissolution rates (kH) for CIP-GA 10% and CIP-GA 25% were significantly higher than those of the SD-CIP control (Figure 3). Due to the poor correlation between CIP-GA 75% and the Higuchi prediction model, this formulation was not considered a matrix system, and its kH value was not calculated.

The nearly 10-fold increase in CIP dissolution capacity observed with CIP-GA 25% microspheres highlights their potential for more effective treatment, particularly given CIP’s poor solubility at lung pH (pH 7.4). This improvement is supported by the Higuchi dissolution rate constants (kH), which demonstrate that both CIP-GA 10% and CIP-GA 25% formulations significantly outperformed SD-CIP, CIP-GA 50%, and CIP-GA 90% in kH values. However, it is important to note that this improvement was only observed when the formulation contained at least 75% GA (Figure 2). The dissolution rate of CIP in the case of CIP-GA 25% increased 8-fold compared to SD-CIP. Conversely, formulations containing 50% or less GA did not improve the dissolution rate of CIP and, in some cases, even slightly decreased it, as observed with CIP-GA 75%.

**Figure 4:**
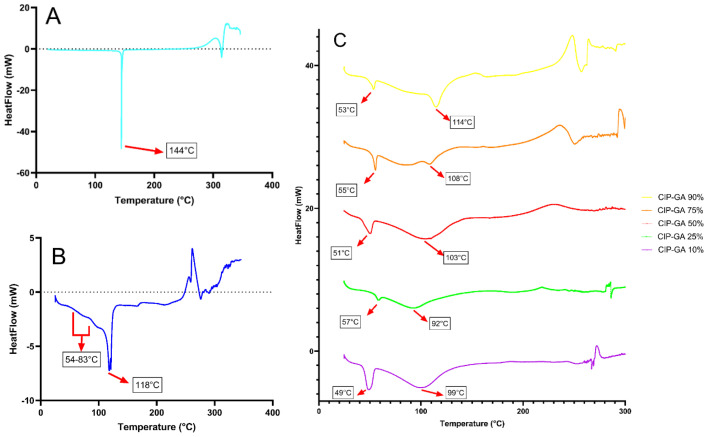
DSC thermograms of CIP formulations. (**A**) CIP HCl; (**B**) SD-CIP; (**C**) CIP-GA microspheres. All spray-dried formulations exhibited a baseline shift compared to CIP HCl, indicative of a glass transition temperature (Tg). The dehydration peak, occurring after the Tg event, progressively shifted to lower temperatures as the GA percentage increased in the formulation.

The DSC thermograms of CIP hydrochloride (CIP HCl) (Figure 4A) exhibited an endothermic peak at 144 °C, corresponding to dehydration, followed by another endothermic peak at 314 °C attributed to CIP’s fusion, and decomposition patterns beginning at 320 °C. For SD-CIP (Figure 4B), a baseline shift was observed between 54 °C and 83 °C, indicating the presence of a glass transition temperature (Tg). This was accompanied by the dehydration endothermic peak shifting to a lower temperature of 118 °C compared to CIP HCl. CIP-GA formulations demonstrated a gradual displacement of the dehydration endothermic peak to lower temperatures when increasing the amount of GA (Figure 4C). Notably, all CIP-GA formulations consistently displayed a Tg as indicated by a slight endothermic peak and a baseline shift preceding the dehydration peak.

The enhancement in CIP dissolution is attributed to the amorphization of the drug, as demonstrated by the DSC. A plausible explanation for the favorable outcomes of CIP-GA 10% and CIP-GA 25% in the dissolution studies (Figure 2) is the formation of an ASD between CIP and GA after the spray drying. Amorphization can occur when drugs are spray-dried either alone or in the presence of polymers; however, the inclusion of polymers plays a crucial role in stabilizing the amorphous state and preventing recrystallization [31]. The DSC scans showed the presence of Tg in all samples containing spray-dried CIP, which is characteristic of ASDs (Figure 4). This hypothesis aligns with previously reported data by Lim et al. [32] and Pas et al. [7], who demonstrated that gelatin could serve as a vehicle for poorly water-soluble drugs, enhancing their dissolution and surpassing their crystalline counterparts. The variation in dissolution profiles among formulations may result from GA’s ability to prevent the recrystallization of CIP by stabilizing it in an amorphous state. This stabilization occurs as GA interposes between the CIP molecules during the rapid drying phase of the spray-drying process, preventing their transition to a more thermodynamically stable crystalline state. The DSC thermograms also evidenced the movement of the dehydration peak of all samples progressively towards lower temperatures as GA percentages in the formulation increased. This phenomenon could correspond to the properties of the ASD formed. ASD states are characterized as being more reactive, as the cohesive interparticle forces are lower than those of crystalline states. Higher amounts of GA in the formulation could form better ASDs by spreading apart the CIP molecules more evenly when they are being dried out, which would lead to a more reactive solid state, which is why the DSC scans show that lower temperatures are required to dehydrate these samples. By maintaining CIP in its amorphous state, GA ensures that CIP retains its enhanced solubility profile over time. Furthermore, the hydrophilic nature of GA promotes water penetration into the matrix, improving the wettability and dissolution of CIP. This synergistic effect—amorphization stabilized by gelatin and improved water interaction—collectively contributes to the enhanced solubility.

The absence of the dissolution enhancement in formulations with 50% or less gelatin (Figure 2) might be attributed to the lower likelihood of CIP dispersing within the GA matrix when co-atomized at lower polymer proportions. As GA likely acts as a dispersing and stabilizing agent, the high CIP content in the 90% formulation may have exceeded the capacity of GA to prevent drug aggregation or recrystallization. Consequently, partial or full recrystallization over time could have occurred, leading to reduced solubility and, in turn, decreased bioavailability and antimicrobial efficacy in vitro. Similar results were observed in the study by Pas et al. [7], where increases in the percentage of loaded active led to a loss of amorphous state in some formulations, directly impacting the dissolution displayed by these formulations. Their findings led them to determine that the increase in amorphous content was directly related to the enhancement of initial and sustained dissolution. They concluded that different interactions between the active ingredients and gelatin likely promoted the amorphous nature of each formulation. Our results also correlate with their conclusions, as the lowest dehydration peaks at 99 °C and 92 °C correspond to the formulations with the highest dissolution: CIP-GA 10% and CIP-GA 25%, respectively.

**Figure 5:**
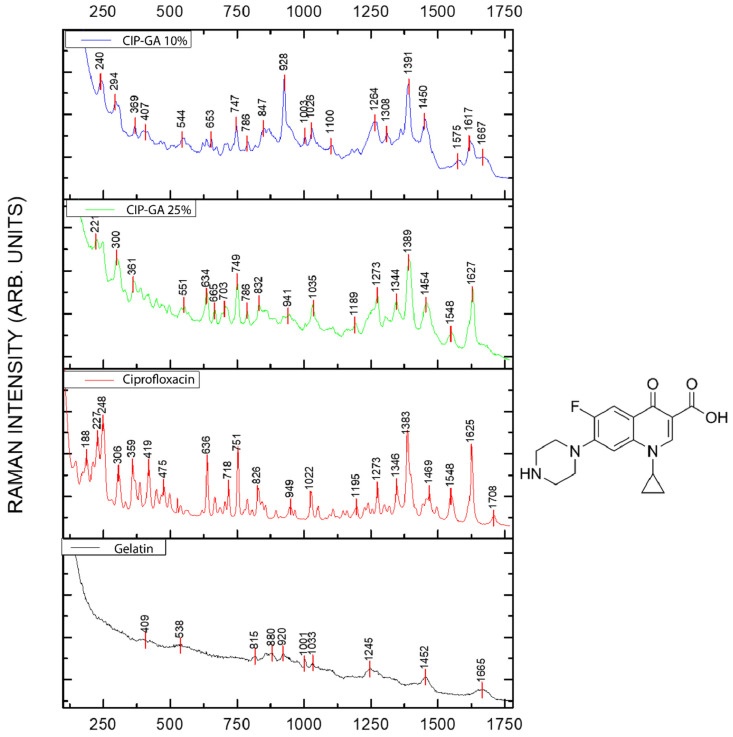
Raman spectra of CIP-GA microspheres and their constituents. The spectra include gelatin (GA), ciprofloxacin (CIP), and the microsphere formulations CIP-GA 10% and CIP-GA 25%. The percentage represents the fraction of the drug in the microsphere. On the right, the chemical structure of CIP is displayed.

On the other hand, to complement the solid-state analysis of the ASD formulations, a Raman spectroscopy study was performed on the crystalline CIP, GA, and microsphere formulations (Figure 5). The formulations with the highest dissolution rates, specifically CIP-GA 10% and CIP-GA 25%, were analyzed by Raman spectroscopy to identify potential interactions between CIP and GA. Figure 5 shows a general broadening of the bands corresponding to the CIP-GA microsphere spectra. Tables S1 and S2 in the Supplementary Materials summarize the reported assignment of the vibrational frequencies for CIP [33,34] and for GA [35,36].

The spectra of CIP-GA microspheres are primarily influenced by the bands associated with CIP. A notable displacement occurs for the band at 1383 cm^−1^ in the CIP spectra, assigned to out-of-plane CH_2_ wagging. As GA increases in the microsphere formulations, this band progressively shifts to a higher frequency, observed at 1389 and 1391 cm^-1^ for CIP-GA 25% and CIP-GA 10%, respectively. Meanwhile, the group of bands around 1469 cm^−1^ is also related to the CH_2_ fragment shifts in the opposite direction to the lower frequency at 1454 and 1450 cm^−1^, converging into a single signal with increased GA content. Some bands, such as the one centered at 1273 cm^−1^, assigned to a CH_2_ twist, are less sensitive to the increase in GA content. This vibration results from multiple contributions, which for some authors include ν(C-F) stretching [36]. This band experiences a shift to a lower frequency by 9 cm^−1^ (1264 cm^−1^) only in the presence of high GA content. Furthermore, the band centered at 949 cm^−1^ on the spectrum of the pure compound also progressively shifts to a lower frequency at 941 and 928 cm^−1^ with the increase in GA content. The bands at 718, 665, and 638 cm^−1^ in the pure CIP compound, which are assigned to =C-H deformation vibrations, as well as the bands around 771 cm^−1^, characteristic of the substituted benzene ring deformation modes, show modifications in both frequency and bandwidth in the CIP-GA microsphere formulations, consistent with an interaction that alters the environment of CIP.

The set of signals from the spectrum of GA has a lower intensity than the pure CIP. However, the bands at 1665, 1452, and 1245 cm^−1^, assigned to amide I vibration, CH_3_-CH_2_ deformation, and amide III vibration, respectively, stand out. These bands overlap with the CIP spectrum without significant shifts, which rules out structural changes like drug degradation that could occur during the microsphere manufacturing process. Analyzing the spectral changes resulting from the interaction between CIP and GA necessitates normal coordinate calculations for isolated and mixed systems. These calculations are essential for a detailed understanding of the microencapsulation process; however, they fall outside the scope of this work. Finally, the Raman spectrum of GA shows characteristic vibrations that agree with those reported in the literature [35,36], as shown in Table S2 (Supplementary Materials).

The displacement of the characteristic bands of the pure drug (CIP) from 1382 cm^−1^ to 1389 and 1391 cm^−1^, and the 1469 cm^−1^ band to 1454 and 1450 cm^−1^, showed a progressive displacement that correlates to the amorphization of CIP as the GA content increases. Moreover, most peaks broadened when the formulations presented more than 75% gelatin, reflecting the interaction between CIP and GA. Similar results have been reported in the literature when analyzing crystal-to-amorphous transitions. Ziaee and coworkers [37] found similar displacements in the bands when analyzing the amorphization process of ibuprofen with the polymer hydroxypropyl methylcellulose acetate succinate (HPMCAS) at different ratios. Raman scattering allows us to visualize how the different regions of the CIP molecule scatter light particles. Thus, the displacement of bands associated with the same molecular fragments between the pure drug (CIP) and CIP-GA microspheres can be attributed to the interaction between CIP and GA. Meanwhile, the bands at 1665, 1452, and 1245 cm^−1^, associated with the gelatin molecular fragments amide I, CH_3_-CH_2_, and amide II, remain unchanged on the microsphere spectra, which rules out structural modifications due to the manufacturing process.

While additional studies may be needed to fully understand the true nature of the CIP-GA microsphere structure, these results clearly demonstrate the potential of this technique for creating formulations aimed at local pulmonary action using biopharmaceutically classified (BCS) type IV drugs, which have low solubility and low permeability. This approach enables the modulation of drug dissolution to enhance efficacy and extend drug action, while preserving their retention due to the inherently limited permeability of the active compounds. 

### 2.3. Gelatin Encapsulation of CIP Increased Biofilm Eradication Efficiency

**Figure 6:**
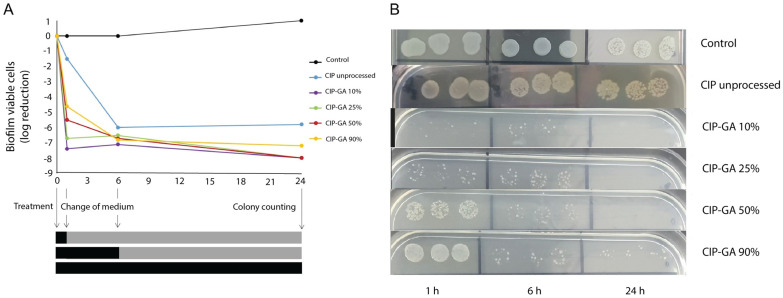
*Pseudomonas aeruginosa* biofilm eradication activity of CIP-GA microspheres. Biofilms were treated with unprocessed CIP and CIP-GA microspheres (10, 25, 50, and 90% of CIP). (**A**) Logarithmic reduction in viable counts (CFU/mL) of dispersed biofilms compared to the negative control. Black bars at the bottom indicate the period with treatment, while gray bars indicate the untreated period (*n* = 3; mean). (**B**) Representative images of viable counts recovered on LB agar after plating the dispersed biofilms.

CIP HCl (unprocessed CIP) and CIP-GA formulations significantly reduced the survival of *P. aeruginosa* biofilms compared to the untreated control (black curve, Figure 6A). After 6 h of treatment, all CIP formulations demonstrated comparable reductions in *P. aeruginosa* viability, achieving log reductions between −6 and −7. This effect was sustained by CIP HCl and CIP-GA 90% even after 24 h.

Interestingly, a distinct gradient in eradication efficacy is observed at 1 h of treatment, inversely proportional to CIP content. CIP HCl achieved a modest reduction of approximately 1 log in surviving biofilm colonies, while CIP-GA 90% resulted in a more pronounced reduction exceeding 4 logs. CIP-GA 10% demonstrated the most potent effect, achieving near-complete eradication within this timeframe. This gradient in biofilm eradication is further illustrated in Figure 6B, depicting the surviving colonies of dispersed biofilms. Formulations with lower CIP content (10, 25, and 50%) eradicated the biofilms completely after 24 h, with no viable colonies observed on the agar plates. In contrast, while unprocessed CIP and CIP-GA 90% substantially reduced viability, they failed to fully eradicate biofilms within the 24 h period.

Given the promising results from the dissolution studies of the CIP-GA formulations, our efforts were directed toward characterizing the time-kill kinetics of the antimicrobial formulations compared to unprocessed CIP HCl. Over time, biofilm eradication studies confirmed the enhanced efficacy of CIP-GA when formulated with 75% or more gelatin.

CIP is a highly potent antibiotic due to its mechanism of action, requiring concentrations as low as 0.1 to 16 µg/mL to inhibit or eradicate *P. aeruginosa* biofilms [38]. According to the Franz cell results, the dissolution of CIP was <1% and 2% for SD-CIP and above 13% and 28% for CIP-GA 25% at 1 and 6 h, respectively. The formulations were tested on biofilms at 125 µg/mL; hence, the dissolution values indicate that the CIP concentrations were approximately 0.8 and 1.6 µg/mL for CIP HCl and 11.2 and 23.2 µg/mL for CIP-GA 25% at 1 and 6 h, respectively—all within or exceeding the eradication concentrations.

Figure 6A shows the changes in the number of viable biofilm CFUs recovered after 1, 6, and 24 h of treatment. A clear gradient in the CIP inhibitory activity can be observed after 1 h of treatment, achieving a higher inhibition with CIP-GA with the lower CIP content. CIP-GA 10% and CIP-GA 25% reached the highest inhibition after only 1 h of treatment, complete eradication after 24 h of treatment (Figure 6B), and the highest CIP release according to Franz cells (Figure 2), indicating that an improvement in CIP release rate correlated with enhanced and faster eradication, even though all formulations achieved equivalent effects in biofilm eradication at 6 h.

CIP amorphization is a solid-state modification that does not involve chemical alteration of the drug molecule. Therefore, the CIP-GA formulations are expected to retain the intrinsic antibacterial spectrum of native CIP, which includes a broad range of Gram-negative and some Gram-positive bacteria. The selection of *P. aeruginosa* as the model pathogen is based on its clinical relevance in chronic respiratory infections, particularly in individuals with CF. *P. aeruginosa* is among the most prevalent and persistent pathogens isolated from the airways of CF patients, and is strongly associated with disease progression, antibiotic resistance, and pulmonary decline [38,39,40,41,42]. Furthermore, its ability to form biofilms represents a key pathogenic mechanism, as biofilm-associated bacteria exhibit significantly increased tolerance to antimicrobial agents and host immune responses [43,44,45]. Given that *P. aeruginosa* primarily exists in biofilm form in the CF lung environment, evaluating the efficacy of our formulation against established *P. aeruginosa* biofilms provides a clinically relevant and stringent model to assess potential therapeutic performance. Biofilms demonstrate notable resistance to antimicrobials compared to planktonic (free-floating) bacteria, partially attributed to the antibiotic tolerance of a subpopulation known as persisters [11,46,47]. Persister cells (phenotypic adaptations) survive by entering a minimal metabolic or dormant state, differing from resistant cells (genotypic adaptations). CIP is known to induce cell death in biofilms while also triggering persistence in surviving cells, and exposure to subinhibitory concentrations can contribute to mutant selection and resistance [48,49,50,51].

Counterintuitively, we found that the formulation with higher CIP content (CIP-GA 90%) failed to eradicate the *P. aeruginosa* biofilms after 24 h of treatment (Figure 6B). In fact, we found that CIP-GA microspheres with lower CIP content (10, 25, and 50%) successfully eradicated the biofilms after 24 h by exerting an enhanced inhibitory effect after only 1 h. Soares et al. [51] reported that phenotypic adaptations in *P. aeruginosa* to CIP can appear after 1 h of treatment, and once a persistent phenotype is expressed, CIP eradication fails. These adaptations may be responsible for the unchanged eradication observed between 6 and 24 h of CIP-GA 90% treatment. In the context of limited antimicrobial development and increased microbial resistance, formulating alternatives that allow for quickly achieving inhibitory concentrations may decrease the induction of persistence and enhance infection eradication. This 1 h timeframe of enhanced dissolution rate and subsequential biofilm eradication is also critical from a biopharmaceutical point of view, as it has been studied that 50–75% of inhaled microparticles can be cleared out from the airways by alveolar macrophages within 2–3 h, underscoring the importance of early efficacy [52]. Therefore, the increase in eradication achieved by our CIP-GA microsphere formulations can contribute to decreasing the amount of CIP available in the environment, increasing the efficiency of the treatment overall.

### 2.4. Gelatin Encapsulation Improved the Aerodynamic Efficiency of the Formulations

The gelatin-based microspheres loaded with 25% CIP (CIP-GA 25%) were selected for the evaluation of morphology, size distribution, and aerodynamic characteristics due to their higher encapsulation efficiency, enhanced dissolution rate, and biofilm eradication efficiency.

**Figure 7:**
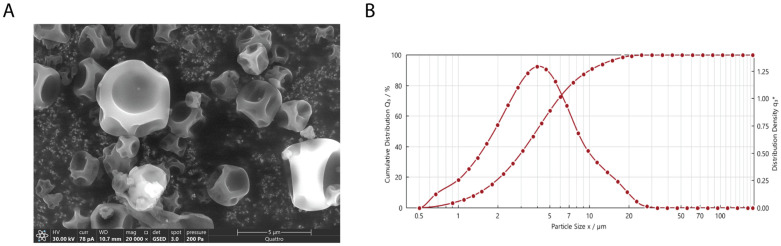
Characterization of CIP-GA 25% microspheres. (**A**) Surface morphology of CIP-25% microspheres obtained at 20,000× magnification. Scale bar represents 5 µm. (**B**) Laser diffraction profile (dry dispersion). Cumulative distribution Q_3_ (left axis) shows the percentage of particles smaller than a specific diameter while the distribution density q_3_* (right axis) represents the probability of finding a particle of a specific diameter.

The particles exhibited spherical morphology with surface concavities, consistent with partial collapse of the particle shell due to capillary forces during the late drying stage (Figure 7A). This is characteristic of hollow or semi-hollow structures where rapid solvent evaporation leads to forming a rigid outer shell that subsequently wrinkles. The laser diffraction profile revealed a D_10_ of 1.44 µm, D_50_ of 3.52 µm, and D_90_ of 10.15 µm, with ~60% of particles under 5 µm, within the optimal range for deep lung deposition (Figure 7B).

**Figure 8:**
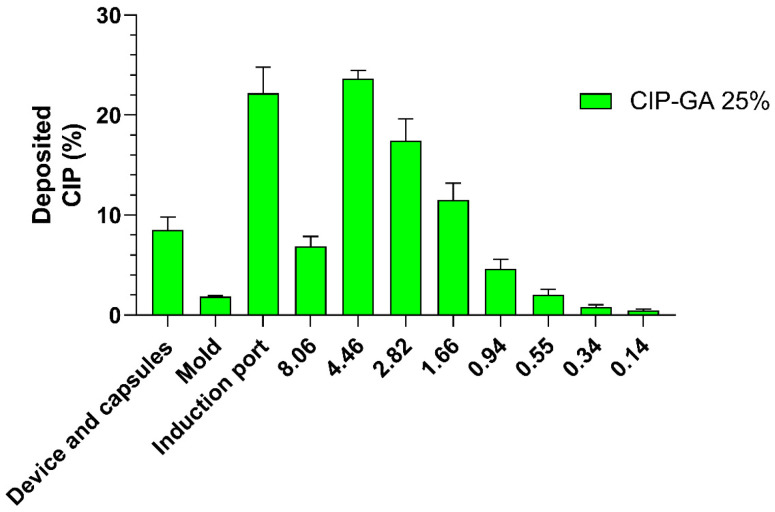
Aerodynamic characterization of the leading CIP-GA formulation. CIP-GA 25% was developed through spray drying, and its aerodynamic performance was assessed through Next-Generation cascade impaction. Each bar represents the %CIP that effectively reaches each stage of the impactor with respect to the total mass loaded on the inhalation device (*n* = 3; mean ± S.D.).

The efficiency of an inhaled drug product can be determined based on its aerodynamic characterization. The key parameters used to evaluate the performance of a DPI are the fine particle fraction (FPF), mass medium aerodynamic diameter (MMAD), and respirable fraction (RF). The CIP-GA 25% formulation showed an FPF of approximately 70% (67.3 ± 2.7%) (Figure 8). The MMAD was 3.60 ± 0.09 μm, and the RF was 60.5 ± 3.2%.

SEM revealed that the CIP-GA 25% spray-dried particles exhibited predominantly spherical morphology, with notable surface indentations and partially collapsed structures (Figure 7A). This morphology is characteristic of hollow or semi-hollow particles formed by rapid shell solidification and structural buckling during drying [53,54]. This structure is expected to improve aerosol performance by reducing particle density and enhancing dispersibility due to the increased surface roughness. Consistently, the aerodynamic studies demonstrated the high deposition performance of the microspheres (Figure 8). The MMAD was measured at 3.6 μm, which increases the likelihood of reaching the alveolus (>0.1 μm and <5 μm). The FPF and RF values obtained in this study for CIP-GA 25% were 67.3 ± 2.7% and 60.5 ± 3.2%, respectively. The small difference between these parameters is an important quality attribute that reflects the good match between the formulation and the device. An ideal device should focus on the emitted powder’s aerodynamic properties and the quantity released from the capsule. In this case, nearly 90% of the drug is emitted, with 60% of the antibiotic reaching the deep lung. These findings are significant compared to the aerosol performance of marketed DPI products documented in the literature, with FPF values typically ranging between 20% and 50% [55,56,57,58]. On the other hand, RF values are often unreported, probably due to the high powder retention in the capsule. Consequently, the CIP-GA microsphere formulation outperforms other products regarding aerodynamic performance. The particle size distribution of the CIP-GA 25% formulation, as determined by laser diffraction, revealed a D_10_ of 1.44 µm, D_50_ of 3.52 µm, and D_90_ of 10.15 µm (Figure 7B). These values are in strong agreement with the mass median aerodynamic diameter (MMAD) of 3.6 µm obtained from Next-Generation Impactor (NGI) measurements, thereby validating the aerodynamic assessment of the formulation. This consistency across measurement methods highlights the high performance of the dry powder, particularly for pulmonary delivery. Furthermore, the laser diffraction data indicated that nearly 60% of the cumulative particle size distribution consisted of particles below 5 µm, a critical threshold for effective deep lung deposition. Thus, the MMAD was substantiated by both indirect laser diffraction analysis and high-resolution SEM imaging, underscoring the robust aerodynamic efficiency of the CIP-GA microspheres (Figure 8).

On the other hand, the high EE (>70%) achieved by all formulations demonstrates that spray drying is an optimal technique to produce inhalable microspheres (Figure 1), outperforming other techniques used in the literature while remaining a one-step, scalable alternative [59,60,61,62]. Thus, this technology holds great potential for advancing into the localized drug delivery of antimicrobials in chronic lung infections, where patients require high and frequent dosing to fight these persistent infections, such as those affecting CF patients.

## 3. Conclusions

We developed an inhalable platform for poorly soluble drugs using gelatin type A, an FDA-approved polymer, to enhance the poor dissolution of ciprofloxacin at lung pH. We identified the best drug–polymer ratio to maximize solubility and the in vitro efficacy of the drug. This formulation significantly improved the antimicrobial efficacy of ciprofloxacin against *P. aeruginosa* biofilms and sustained its release for at least 6 h. The aerodynamic performance of our platform was highly efficient, surpassing the currently available options on the market. Overall, this study presents a promising formulation alternative for reformulating poorly dissolving drugs and antibiotics for localized lung delivery.

## 4. Materials and Methods

### 4.1. Materials

Ciprofloxacin was used in the form of its hydrochloride salt conjugate (CIP HCl) (AK Scientific, Inc., Union City, CA, USA), along with type A gelatin (Sigma Aldrich, St. Louis, MO, USA). Merck KGaA (Darmstadt, Germany) provided the potassium dihydrogen phosphate and sodium hydroxide, as well as the culture media (Luria–Bertani broth and agar) and phosphate-buffered saline (PBS). The strain of *Pseudomonas aeruginosa* (ATCC 27853) was provided by Microbiologics, Inc. (St. Cloud, MN, USA). Other reagents used in this research were acetic acid (CDH, New Delhi, India), sodium acetate anhydrous AR/ACS (CDH, New Delhi, India), and size 3 gelatin capsules (Capsugel Lonza, Morristown, NJ, USA)

### 4.2. Development of CIP-Loaded GA Microspheres

All feeding solutions were prepared with a consistent solid concentration of 1% (*w*/*v*). Five formulations with varying ratios of CIP and GA were produced (CIP-GA) to assess the effect of polymer concentration on the dissolution rate of CIP and its amorphization within the resulting microspheres. Formulations were labeled based on their CIP content percentage (Table 1). The solids in the feeding solution were dissolved in a pH 4.5 acetate buffer at 45 °C under constant stirring to ensure the complete dissolution of both components. These conditions were maintained throughout the drying process. Spray-drying parameters were set according to a Box–Behnken Design of Experiments (DoE) to optimize the aerosol performance of gelatin microspheres established in a previous study by Behrend-Keim et al. [25]. Briefly, the feed rate was set at 3 mL/min, the inlet temperature was 120 °C, and the atomization flow was 1050 L/h, using a two-fluid nozzle with a Büchi Mini Spray dryer B-290 (Büchi, Labortechnik AG, Flawil, Switzerland). Dry powders were recovered from the collector vessel and immediately stored in a desiccator. As a control, a feeding solution of CIP HCl 1% (*w*/*v*) was prepared using pH 4.5 acetate buffer as a solvent, and the spray-drying process was carried out as described above. 

### 4.3. Encapsulation Efficiency (EE)

%EE was defined as the ratio of the actual amount of CIP contained in the microspheres to the expected (theoretical) amount of CIP based on the feeding solutions, expressed as a percentage. The assessment was performed in triplicate by dissolving a known quantity of the formulation in acetate buffer (pH 4.5) at 37 °C for 24 h to ensure maximum drug dissolution. CIP was quantified by UV spectroscopy, as described below. The %EE was calculated using the following equation:

(1) $$Encapsulation\;efficiency=\left(\frac{Experimental\;CIP\;in\;the\;weighted\;mass}{Theoretical\;CIP\;in\;the\;weighted\;mass}\right)\times 100$$

### 4.4. Drug Quantification

CIP was quantified using UV spectroscopy (Synergy H1M, Biotek Instruments, Santa Clara, CA, USA) at a wavelength of 350 nm when samples were recovered with acetate buffer at pH 4.5, or 300 nm when samples were collected with phosphate buffer at pH 7.4. To eliminate potential interference from gelatin in the matrix, absorbance measurements were taken at 350 nm rather than at the typical λ_max_ of CIP (271 nm). This adjustment allowed for a clearer and more specific quantification of CIP in the release medium. The spectrophotometric method was validated for linearity and precision within an absorbance range of 0.15 to 0.85, covering the concentration range expected across all experimental settings, including %EE, CIP dissolution, and CIP deposition in the determination of aerodynamic performance. All calibration curves demonstrated strong linearity (R^2^ > 0.998) and excellent repeatability (%RSD < 2%). The complete calibration data and validation results are provided in the Supplementary Information to support reproducibility and transparency.

### 4.5. Dissolution Testing

The dissolution test used in this study followed the methodology developed by Salama and coworkers [63] but used a regular Franz diffusion cell. An amount equivalent to 5 mg of the sample (SD-CIP and CIP-GA formulations) was placed in an MCE membrane filter (0.45 µm) and loaded in a Franz diffusion cell with dissolution medium (0.05 M phosphate buffer, pH 7.4) at 37 °C. Samples were taken at 5, 10, 15, 30, 60, 90, 180, and 360 min to analyze the release profiles. This experiment was performed in quadruplicate. The cumulative percentage of CIP released was calculated based on the total amount of drug recovered. This included dissolving the remaining formulation in the filter with acetate buffer (pH 4.5) for 30 min at 60 °C [64] at the end of the dissolution test, allowing for calculating the total amount of drug present in the experiment. The release profiles of CIP-GA formulations were modeled using Higuchi’s model, since it is ideal for matrix systems. Higher values of the parameter t50(h) and Higuchi’s model release constant (kH) indicate higher release rates. These parameters were calculated using the DDsolver add-in to compare the dissolution profiles between formulations [64]. Finally, statistical differences between kH values were analyzed by ANOVA and Tukey’s HSD test with a *p* value of 0.05 using the software GraphPad Prism 9.0.0 (GraphPad Software LLC, Boston, MA, USA).

### 4.6. Differential Scanning Calorimetry (DSC) Assay

DSC was performed using DSC131 equipment (SETARAM Inc., Cranbury, NJ, USA) to evaluate the thermal properties of the different microsphere formulations with varying percentages of gelatin and CIP. Approximately 5 mg of each formulation was accurately weighed, placed in aluminum DSC pans, and loaded into the DSC instrument. The instrument was programmed to heat the samples from 25 °C to 350 °C at a constant rate of 5 °C/min. During the analysis, heat flow and temperature changes were recorded as a function of time. The resulting DSC thermograms were analyzed to assess the thermal behavior and transitions of the various formulations. Key parameters were identified and documented, including peak temperatures, enthalpy changes, and transition events. DSC thermograms for CIP HCl and type A gelatin were also obtained under the same conditions and used as control samples.

### 4.7. Biofilm Eradication Efficacy of CIP Microspheres

*Pseudomonas aeruginosa* (ATCC 27853) biofilms were grown for 24 h, as described by Bahamondez-Canas et al. [65]. Briefly, about five homogeneous colony-forming units (CFUs) were used to inoculate 10 mL of Luria–Bertani (LB) medium. After overnight incubation at 180 rpm and 37 °C, the bacterial concentration was adjusted to 3 × 10^7^ CFU/mL using a spectrophotometer at an optical density of 625 nm (Synergy H1M, Biotek Instruments, Santa Clara, CA, USA), and 1000 μL was added to the wells of a 12-well plate, which was then sealed and incubated for 1.5 h at 75 rpm and 37 °C on an orbital shaker. Finally, the suspension was discarded, the wells were rinsed with phosphate buffer saline (PBS) to remove non-adherent bacteria, and 3000 μL of fresh LB medium was added to each well before a second incubation at 37 °C and 75 rpm for 24 h.

The media were removed to evaluate the microspheres on the biofilms, and the wells were rinsed with PBS to discard detached cells. For the CIP-GA microsphere treatment, enough formulation was weighed to obtain an amount of CIP equivalent to 125 μg/mL. Microspheres were tested by directly pouring the powder onto the pre-filled wells containing 3 mL of LB medium, as described by Bahamondez-Canas et al. [65]. As a positive control, unprocessed CIP (CIP HCl) was dissolved in acetate buffer at pH 4.5 and then diluted with LB medium to obtain the same CIP concentration (125 μg/mL). Wells filled with 3 mL of LB medium served as negative controls. After the treatments were added, the plates were sealed and incubated at 37 °C and 75 rpm. After 1 and 6 h, the media of the wells were removed, and the wells were washed with PBS to stop the treatment and subsequently filled with fresh culture medium. Then, the plates were sealed and incubated under the same conditions as described previously. After 24 h, the media of all the wells were removed, and the wells were washed and sonicated for 30 min in 3 mL of PBS. Dispersed biofilms were collected in tubes. Each tube with dispersed biofilm was vortexed for 1 min and serially diluted by factors of 10. Then, three drops (10 µL) of each dilution were plated onto LB agar plates using the drop-plate method to determine the viability of bacteria [66]. The results represent the average and standard deviation of three independent experiments and are expressed as the logarithmic changes in CFU/mL between the treated biofilms and the negative control group.

### 4.8. Morphology, Sizing, and Aerodynamic Performance of the CIP-Loaded Microspheres

Micrographs of the CIP-GA 25% powders were collected using a Field-Emission Scanning Electron Microscope (FESEM) (Quattro S, ThermoFisher Scientific Inc., Waltham, MA, USA) with a gaseous detector (GSED) in environmental mode (ESEM).

The particle size distribution was analyzed using a SYMPATEC HELOS-BR laser diffraction particle size analyzer equipped with a RODOS disperser. For dispersion, compressed air was applied at a pressure of 3.4 bar through the RODOS unit to ensure effective deagglomeration without compromising particle integrity. Samples were introduced in quantities sufficient to ensure representative analysis while avoiding system multiple scattering events, following USP <429> specifications. Measurements were taken in triplicate, and the average values were used for analysis. The system software recorded the particle size distribution, reporting D_50_ (median diameter) as the primary metric. The span, indicating distribution breadth, was calculated as (D_90_ − D_10_)/D_50_. Data are reported based on Q3 measurements, indicating volume-based quantification. To ensure the accuracy of this indirect measurement and confirm that the reported values did not represent agglomerated particles, data were cross-validated with high-resolution imaging obtained via SEM.

The aerodynamic performance of CIP-GA microspheres was assessed in triplicate using a Next-Generation Impactor (NGI, MSP Corporation, Shoreview, MN, USA). Gelatin capsules of size N°3 (Capsugel^®^, Lonza Group, Basel, Switzerland) were loaded with 20 mg of the dry powder formulations. Three capsules of the CIP-GA 25% formulation were aerosolized using a DPI RS01 device (Plastiape^®^, Osnago, Italy) with an airflow of 60 L/min and a pressure drop of 4 KPa. Each NGI stage was coated with a solution of 1% (*w*/*v*) mineral oil in hexane. The particles deposited in each stage of the NGI (and in the inhaler, capsules, inhaler adapter, and induction port) were collected with a defined volume (3 to 15 mL) of acetate buffer, pH 4.5, at 60 °C to promote maximum drug dissolution. The FPF and RF were calculated for the CIP-GA 25% formulation:

(2) $$FPF=\left(\frac{mass\;of\;particles<5\;\mu m}{mass\;of\;emitted\;dose}\right)\times 100$$

(3) $$RF=\left(\frac{mass\;of\;particles<5\;\mu m}{total\;mass\;loaded\;on\;the\;device}\right)\times 100$$

FPF and RF were used to determine the aerodynamic efficiency of the microspheres. The formulation MMAD (μm) and fine particle dose (FPD, µg or mg) were also determined.

### 4.9. Raman Spectroscopy

To obtain the Raman spectra, a sample of the formulations and their independent constituents was placed on a glass slide, and the spectrum was subsequently recorded using an Alpha 300 SNOM-Raman microscope (WITec GmbH, Ulm, Germany) equipped with a 785 nm excitation laser line and an electrically cooled charge-coupled device (CCD) detector. The signal was calibrated using the silicon line at 520 cm^−1^, employing a monocrystalline Si sheet and a 20× objective. The laser power on the samples was 2 mW. The resolution was set to 4 cm^−1^, and 10 scans were performed with an integration time of 1 s. The spectra were recorded in the range between 200 and 1800 cm^−1^. The independent spectrum and the spectrum of the formulation with the highest dissolution rate were analyzed to determine the interaction between CIP and GA by analyzing peak displacement and broadening.

### 4.10. Statistical Analysis

All results are presented as mean ± standard deviation (S.D.). Dissolution rates, derived from dissolution profile data, were analyzed using one-way analysis of variance (ANOVA), followed by Tukey’s Honest Significant Difference (HSD) post hoc test for multiple comparisons using GraphPad Prism 9.0.0 (GraphPad Software LLC, Boston, MA, USA). A *p*-value of less than 0.05 was considered indicative of statistical significance.

## Figures and Tables

**Table 1:** CIP-GA microspheres developed by spray drying.

Formulation	CIP:GA Ratio
CIP-GA 90%	9:1
CIP-GA 75%	3:1
CIP-GA 50%	1:1
CIP-GA 25%	1:3
CIP-GA 10%	1:9

## Data Availability

The raw data supporting the conclusions of this article will be made available by the authors on request.

## Supplementary Materials

The following supporting information can be downloaded at: https://www.mdpi.com/article/10.3390/gels11080567/s1, Table S1: Ciprofloxacin vibrational assignments; Table S2: Gelatin vibrational assignments.

## Abbreviations

The following abbreviations are used in this manuscript:

ASD	Amorphous solid dispersion
CIP	Ciprofloxacin
CF	Cystic fibrosis
CFU	Colony-forming units
DPI	Dry powder inhaler
ED	Emitted dose
FPD	Fine particle dose
FPF	Fine particle fraction
GA	Gelatin type A
LB	Luria–Bertani culture media
MMAD	Mass medium aerodynamic diameter
NGI	Next-generation impactor

## References

[1] Gbian D.L., Omri A. (2021). Current and Novel Therapeutic Strategies for the Management of Cystic Fibrosis. Expert Opin. Drug Deliv..

[2] Jackson A.D., Goss C.H. (2018). Epidemiology of CF: How Registries Can Be Used to Advance Our Understanding of the CF Population. J. Cyst. Fibros..

[3] Scotet V., L’hostis C., Férec C. (2020). The Changing Epidemiology of Cystic Fibrosis: Incidence, Survival and Impact of the CFTR Gene Discovery. Genes.

[4] Rowe S.M., Stacey M., Sorscher E.J. (2024). Cystic Fibrosis. N. Engl. J. Med..

[5] Cohen-Cymberknoh M., Shoseyov D., Kerem E. (2011). Managing Cystic Fibrosis: Strategies That Increase Life Expectancy and Improve Quality of Life. Am. J. Respir. Crit. Care Med..

[6] Alhajj N., O’Reilly N.J., Cathcart H. (2022). Developing Ciprofloxacin Dry Powder for Inhalation: A Story of Challenges and Rational Design in the Treatment of Cystic Fibrosis Lung Infection. Int. J. Pharm..

[7] Pas T., Vergauwen B., Van den Mooter G. (2018). Exploring the Feasibility of the Use of Biopolymers as a Carrier in the Formulation of Amorphous Solid Dispersions–Part I: Gelatin. Int. J. Pharm..

[8] Kapourani A., Vardaka E., Katopodis K., Kachrimanis K., Barmpalexis P. (2020). Crystallization Tendency of APIs Possessing Different Thermal and Glass Related Properties in Amorphous Solid Dispersions. Int. J. Pharm..

[9] Pas T., Struyf A., Vergauwen B., Van den Mooter G. (2018). Ability of Gelatin and BSA to Stabilize the Supersaturated State of Poorly Soluble Drugs. Eur. J. Pharm. Biopharm..

[10] Maurice N.M., Bedi B., Sadikot R.T. (2018). Pseudomonas Aeruginosa Biofilms: Host Response and Clinical Implications in Lung Infections. Am. J. Respir Cell Mol. Biol..

[11] Drenkard E., Ausubel F.M. (2002). Pseudomonas Biofilm Formation and Antibiotic Resistance Are Linked to Phenotypic Variation. Nature.

[12] Doring G., Conway S.P., Heijerman H.G.M., Hodson M.E., Hoiby N., Smyth A., Touw D.J. (2000). Antibiotic Therapy against Pseudomonas Aeruginosa in Cystic Fibrosis: A European Consensus. Eur. Respir. J..

[13] Mayer-Hamblett N., Kloster M., Rosenfeld M., Gibson R.L., Retsch-Bogart G.Z., Emerson J., Thompson V., Ramsey B.W. (2015). Impact of Sustained Eradication of New Pseudomonas Aeruginosa Infection on Long-Term Outcomes in Cystic Fibrosis. Clin. Infect. Dis..

[14] Shariati A., Arshadi M., Khosrojerdi M.A., Abedinzadeh M., Ganjalishahi M., Maleki A., Heidary M., Khoshnood S. (2022). The Resistance Mechanisms of Bacteria against Ciprofloxacin and New Approaches for Enhancing the Efficacy of This Antibiotic. Front. Public Health.

[15] Serisier D.J., Bilton D., De Soyza A., Thompson P.J., Kolbe J., Greville H.W., Cipolla D., Bruinenberg P., Gonda I. (2013). Inhaled, Dual Release Liposomal Ciprofloxacin in Non-Cystic Fibrosis Bronchiectasis (ORBIT-2): A Randomised, Double-Blind, Placebo-Controlled Trial. Thorax.

[16] Haworth C.S., Bilton D., Chalmers J.D., Davis A.M., Froehlich J., Gonda I., Thompson B., Wanner A., O’Donnell A.E. (2019). Inhaled Liposomal Ciprofloxacin in Patients with Non-Cystic Fibrosis Bronchiectasis and Chronic Lung Infection with Pseudomonas Aeruginosa (ORBIT-3 and ORBIT-4): Two Phase 3, Randomised Controlled Trials. Lancet Respir. Med..

[17] Golomb B.A., Koslik H.J., Redd A.J. (2015). Case Report: Fluoroquinolone-Induced Serious, Persistent, Multisymptom Adverse Effects. BMJ Case Rep..

[18] Varanda F., Pratas de Melo M.J., Caço A.I., Dohrn R., Makrydaki F.A., Voutsas E., Tassios D., Marrucho I.M. (2006). Solubility of Antibiotics in Different Solvents. 1. Hydrochloride Forms of Tetracycline, Moxifloxacin, and Ciprofloxacin. Ind. Eng. Chem. Res..

[19] Caço A.I., Varanda F., Pratas de Melo M.J., Dias A.M.A., Dohrn R., Marrucho I.M. (2008). Solubility of Antibiotics in Different Solvents. Part II. Non-Hydrochloride Forms of Tetracycline and Ciprofloxacin. Ind. Eng. Chem. Res..

[20] Park H.-R., Kim T.H., Bark K.-M. (2002). Physicochemical Properties of Quinolone Antibiotics in Various Environments. Eur. J. Med. Chem..

[21] Ross D.L., Riley C.M. (1990). Aqueous Solubilities of Some Variously Substituted Quinolone Antimicrobials. Int. J. Pharm..

[22] Karimi K., Katona G., Csóka I., Ambrus R. (2018). Physicochemical Stability and Aerosolization Performance of Dry Powder Inhalation System Containing Ciprofloxacin Hydrochloride. J. Pharm. Biomed. Anal..

[23] Shetty N., Zeng L., Mangal S., Nie H., Rowles M.R., Guo R., Han Y., Park J.H., Zhou Q. (2018). Effects of Moisture-Induced Crystallization on the Aerosol Performance of Spray Dried Amorphous Ciprofloxacin Powder Formulations. Pharm. Res..

[24] Shetty N., Park H., Zemlyanov D., Mangal S., Bhujbal S., Zhou Q.T. (2018). Influence of Excipients on Physical and Aerosolization Stability of Spray Dried High-Dose Powder Formulations for Inhalation. Int. J. Pharm..

[25] Behrend-Keim B., Castro-Muñoz A., Monrreal-Ortega L., Ávalos-León B., Campos-Estrada C., Smyth H.D.C., Bahamondez-Canas T.F., Moraga-Espinoza D. (2023). The Forgotten Material: Highly Dispersible and Swellable Gelatin-Based Microspheres for Pulmonary Drug Delivery of Cromolyn Sodium and Ipratropium Bromide. Int. J. Pharm..

[26] U.S. Food and Drug Administration (FDA) Inactive Ingredient Database. https://www.Accessdata.Fda.Gov/Scripts/Cder/Iig/Index.Cfm.

[27] Olivera M.E., Manzo R.H., Junginger H.E., Midha K.K., Shah V.P., Stavchansky S., Dressman J.B., Barends D.M. (2011). Biowaiver Monographs for Immediate Release Solid Oral Dosage Forms: Ciprofloxacin Hydrochloride. J. Pharm. Sci..

[28] Marques M.R.C., Loebenberg R., Almukainzi M. (2011). Simulated Biological Fluids with Possible Application in Dissolution Testing. Dissolution Technol..

[29] Hastedt J.E., Bäckman P., Cabal A., Clark A., Ehrhardt C., Forbes B., Hickey A.J., Hochhaus G., Jiang W., Kassinos S. (2022). IBCS: 1. Principles and Framework of an Inhalation-Based Biopharmaceutics Classification System. Mol. Pharm..

[30] Silva D.M., Vyas H.K.N., Sanderson-Smith M.L., Sencadas V. (2018). Development and Optimization of Ciprofloxacin-Loaded Gelatin Microparticles by Single-Step Spray-Drying Technique. Powder Technol..

[31] Singh A., Van den Mooter G. (2016). Spray Drying Formulation of Amorphous Solid Dispersions. Adv. Drug Deliv. Rev..

[32] Lim H.-T., Balakrishnan P., Oh D.H., Joe K.H., Kim Y.R., Hwang D.H., Lee Y.-B., Yong C.S., Choi H.-G. (2010). Development of Novel Sibutramine Base-Loaded Solid Dispersion with Gelatin and HPMC: Physicochemical Characterization and Pharmacokinetics in Beagle Dogs. Int. J. Pharm..

[33] Sinha R.K., Biswas P. (2020). Structural Elucidation of Levofloxacin and Ciprofloxacin Using Density Functional Theory and Raman Spectroscopy with Inexpensive Lab-Built Setup. J. Mol. Struct..

[34] Rajalakshmi K., Gunasekaran S., Kumaresan S. (2014). Vibrational Spectra, Electronic and Quantum Mechanical Investigations on Ciprofloxacin. Indian J. Phys..

[35] Frushour B.G., Koenig J.L. (1975). Raman Scattering of Collagen, Gelatin, and Elastin. Biopolymers.

[36] Liu X., Zhang N., Yu L., Zhou S., Shanks R., Zheng J. (2016). Imaging the Phase of Starch–Gelatin Blends by Confocal Raman Microscopy. Food Hydrocoll..

[37] Ziaee A., O’Dea S., Howard-Hildige A., Padrela L., Potter C., Iqbal J., Albadarin A.B., Walker G., O’Reilly E.J. (2019). Amorphous Solid Dispersion of Ibuprofen: A Comparative Study on the Effect of Solution Based Techniques. Int. J. Pharm..

[38] Hassan M.M., Harrington N.E., Sweeney E., Harrison F. (2020). Predicting Antibiotic-Associated Virulence of Pseudomonas Aeruginosa Using an Ex Vivo Lung Biofilm Model. Front Microbiol..

[39] Pier G.B. (1983). Cystic Fibrosis and Pseudomonas Infection. Lancet.

[40] Morita Y., Tomida J., Kawamura Y. (2014). Responses of Pseudomonas Aeruginosa to Antimicrobials. Front Microbiol..

[41] Fick R.B. (1989). Pathogenesis of the Pseudomonas Lung Lesion in Cystic Fibrosis. Chest.

[42] Ciszek-Lenda M., Strus M., Walczewska M., Majka G., Machul-Żwirbla A., Mikołajczyk D., Górska S., Gamian A., Chain B., Marcinkiewicz J. (2019). Pseudomonas Aeruginosa Biofilm Is a Potent Inducer of Phagocyte Hyperinflammation. Inflamm. Res..

[43] Nixon G.M., Armstrong D.S., Carzino R., Carlin J.B., Olinsky A., Robertson C.F., Grimwood K. (2001). Clinical Outcome after Early Pseudomonas Aeruginosa Infection in Cystic Fibrosis. J. Pediatr..

[44] Werner E., Roe F., Bugnicourt A., Franklin M.J., Heydorn A., Molin S., Pitts B., Stewart P.S. (2004). Stratified Growth in Pseudomonas Aeruginosa Biofilms. Appl. Environ. Microbiol..

[45] Stewart P.S., Costerton J.W. (2001). Antibiotic Resistance of Bacteria in Biofilms. Lancet.

[46] Sauer K., Stoodley P., Goeres D.M., Hall-Stoodley L., Burmølle M., Stewart P.S., Bjarnsholt T. (2022). The Biofilm Life Cycle: Expanding the Conceptual Model of Biofilm Formation. Nat. Rev. Microbiol..

[47] Vulin C., Leimer N., Huemer M., Ackermann M., Zinkernagel A.S. (2018). Prolonged Bacterial Lag Time Results in Small Colony Variants That Represent a Sub-Population of Persisters. Nat. Commun..

[48] Ahmed M.N., Abdelsamad A., Wassermann T., Porse A., Becker J., Sommer M.O.A., Høiby N., Ciofu O. (2020). The Evolutionary Trajectories of P. Aeruginosa in Biofilm and Planktonic Growth Modes Exposed to Ciprofloxacin: Beyond Selection of Antibiotic Resistance. NPJ Biofilms Microbiomes.

[49] Ahmed M.N., Porse A., Sommer M.O.A., Høiby N., Ciofu O. (2018). Evolution of Antibiotic Resistance in Biofilm and Planktonic Pseudomonas Aeruginosa Populations Exposed to Subinhibitory Levels of Ciprofloxacin. Antimicrob. Agents Chemother..

[50] Ołdak E., Trafny E.A. (2005). Secretion of Proteases by Pseudomonas Aeruginosa Biofilms Exposed to Ciprofloxacin. Antimicrob. Agents Chemother..

[51] Soares A., Roussel V., Pestel-Caron M., Barreau M., Caron F., Bouffartigues E., Chevalier S., Etienne M. (2019). Understanding Ciprofloxacin Failure in Pseudomonas Aeruginosa Biofilm: Persister Cells Survive Matrix Disruption. Front Microbiol..

[52] Geiser M. (2010). Update on Macrophage Clearance of Inhaled Micro- and Nanoparticles. J. Aerosol. Med. Pulm. Drug Deliv..

[53] Mudie D.M., Buchanan S., Stewart A.M., Smith A., Shepard K.B., Biswas N., Marshall D., Ekdahl A., Pluntze A., Craig C.D. (2020). A Novel Architecture for Achieving High Drug Loading in Amorphous Spray Dried Dispersion Tablets. Int. J. Pharm. X.

[54] Lechanteur A., Evrard B. (2020). Influence of Composition and Spray-Drying Process Parameters on Carrier-Free DPI Properties and Behaviors in the Lung: A Review. Pharmaceutics.

[55] Palander A., Mattila T., Karhu M., Muttonen E. (2000). In Vitro Comparison of Three Salbutamol-Containing Multidose Dry Powder Inhalers: Buventol Easyhaler^®^, Inspiryl Turbuhaler^®^ and Ventoline Diskus. Clin. Drug. Investig..

[56] Ciciliani A.-M., Langguth P., Wachtel H. (2017). In Vitro Dose Comparison of Respimat^®^ Inhaler with Dry Powder Inhalers for COPD Maintenance Therapy. Int. J. Chron. Obstruct. Pulmon. Dis..

[57] Yildiz-Peköz A., Akbal O., Tekarslan S.H., Sagirli A.O., Mulazimoglu L., Morina D., Cevher E. (2018). Preparation and Characterization of Doripenem-Loaded Microparticles for Pulmonary Delivery. J. Aerosol. Med. Pulm. Drug Deliv..

[58] Demoly P., Hagedoorn P., de Boer A.H., Frijlink H.W. (2014). The Clinical Relevance of Dry Powder Inhaler Performance for Drug Delivery. Respir. Med..

[59] Remanan M.K., Zhu F. (2021). Encapsulation of Rutin Using Quinoa and Maize Starch Nanoparticles. Food Chem..

[60] Sanna V., Roggio A.M., Pala N., Marceddu S., Lubinu G., Mariani A., Sechi M. (2015). Effect of Chitosan Concentration on PLGA Microcapsules for Controlled Release and Stability of Resveratrol. Int. J. Biol. Macromol..

[61] Mohamed F., van der Walle C.F. (2006). PLGA Microcapsules with Novel Dimpled Surfaces for Pulmonary Delivery of DNA. Int. J. Pharm..

[62] Yun P., Devahastin S., Chiewchan N. (2021). Microstructures of Encapsulates and Their Relations with Encapsulation Efficiency and Controlled Release of Bioactive Constituents: A Review. Compr. Rev. Food Sci. Food Saf..

[63] Salama R.O., Traini D., Chan H.-K., Young P.M. (2008). Preparation and Characterisation of Controlled Release Co-Spray Dried Drug–Polymer Microparticles for Inhalation 2: Evaluation of in Vitro Release Profiling Methodologies for Controlled Release Respiratory Aerosols. Eur. J. Pharm. Biopharm..

[64] Zhang Y., Huo M., Zhou J., Zou A., Li W., Yao C., Xie S. (2010). DDSolver: An Add-in Program for Modeling and Comparison of Drug Dissolution Profiles. AAPS J..

[65] Bahamondez-Canas T.F., Ferrati S., Moraga-Espinoza D.F., Smyth H.D.C. (2018). Development, Characterization, and In Vitro Testing of Co-Delivered Antimicrobial Dry Powder Formulation for the Treatment of Pseudomonas Aeruginosa Biofilms. J. Pharm. Sci..

[66] Herigstad B., Hamilton M., Heersink J. (2001). How to Optimize the Drop Plate Method for Enumerating Bacteria. J. Microbiol. Methods.

