# Supplementary material for: Gelatin-Based Microspheres of Ciprofloxacin for Enhanced Lung Delivery and Biofilm Eradication in Pseudomonas aeruginosa Pulmonary Infections
Source: Gels. 2025 Jul 23;11(8):567. doi: 10.3390/gels11080567 (PMC12385393; doi:10.3390/gels11080567)
Supplement: Supplementary file 1 [file gels-11-00567-s001.zip › gels-3639191-supplementary.pdf]

Supplementary material

Table S1. Ciprofloxacin vibrational assignments

**Frequencies exp/ (cm^{-1})**	**Assignments**
1708	νC=O stretch, and one bending in the plane δO–H
1625	νC–C stretch of benzene ring
1548	C=O stretch + C–C stretch in C-N ring
1495	C–H in-plane bend + C–N stretch
1469	CH_2 scissoring
1383	CH_2 wagging
1346	CH_2 wagging + C–C stretch + C–N stretch + O–H in-plane bend
1273	Ring breathing + in-pane C–H bend + CH_2 twist
1024	γ(C–H) β(C–N)
946	γ (C–H) ring
ν, stretching; ν_{sym}, symmetric stretching; ν_{asy}, asymmetric stretching; β, in-plane bending; γ, out-of-plane bending; δ_s, symmetrical deformation; δ_{as}, asymmetrical deformation; ζ, scissoring; ρ, rocking; ρ_i, in-plane rocking; ρ_o, out-of-plane rocking; ω, wagging; τ, twisting, trig-trigonal	

Table S2. Gelatin vibrational assignments

**Frequencies exp/ (cm^{-1})**	**Assignments**
1665	Amide I
1452	$\delta(CH_3, CH_2)$
1245	Amida III
1033	Pro
1001	Phe
920	ν(C-C) Pro ring
880	ν(C-C) Hypro ring
815	ν(C-C) skeletal
538	--- Phe
409	--- Hypro
ν, stretching; ν_{sym}, symmetric stretching; ν_{asy}, asymmetric stretching; β, in-plane bending; γ, out-of-plane bending; δ_s, symmetrical deformation; δ_{as}, asymmetrical deformation; ζ, scissoring; ρ, rocking; ρ_i, in-plane rocking; ρ_o, out-of-plane rocking; ω, wagging; τ, twisting, trig-trigonal	
